# GPR137 Inhibits Cell Proliferation and Promotes Neuronal Differentiation in the Neuro2a Cells

**DOI:** 10.1007/s11064-022-03833-4

**Published:** 2022-11-27

**Authors:** Kensuke Iwasa, Anzu Yamagishi, Shinji Yamamoto, Chikara Haruta, Kei Maruyama, Keisuke Yoshikawa

**Affiliations:** Department of Pharmacology, Faculty of Medicine, Saitama Medical University, 38 Moro-Hongo, Moroyama-Machi, Iruma-Gun, Saitama, 350-0495 Japan

**Keywords:** GPR137, Neuro2A, CRISPR/Cas9, Cell cycle exit, Neuronal differentiation

## Abstract

The orphan receptor, G protein-coupled receptor 137 (GPR137)*,* is an integral membrane protein involved in several types of cancer. *GPR137* is expressed ubiquitously, including in the central nervous system (CNS). We established a *GPR137* knockout (KO) neuro2A cell line to analyze *GPR137* function in neuronal cells. KO cells were generated by genome editing using clustered regularly interspaced short palindromic repeats (CRISPR)/Cas9 and cultured as single cells by limited dilution. Rescue cells were then constructed to re-express *GPR137* in *GPR137* KO neuro2A cells using an expression vector with an EF1-alpha promoter. *GPR137* KO cells increased cellular proliferation and decreased neurite outgrowth (i.e., a lower level of neuronal differentiation). Furthermore, *GPR137* KO cells exhibited increased expression of a cell cycle regulator, cyclin D1, and decreased expression of a neuronal differentiation marker, NeuroD1. Additionally, *GPR137* KO cells exhibited lower expression levels of the neurite outgrowth markers STAT3 and GAP43. These phenotypes were all abrogated in the rescue cells. In conclusion, *GPR137* deletion increased cellular proliferation and decreased neuronal differentiation, suggesting that *GPR137* promotes cell cycle exit and neuronal differentiation in neuro2A cells. Regulation of neuronal differentiation by *GPR137* could be vital to constructing neuronal structure during brain development.

**Graphical Abstract:**

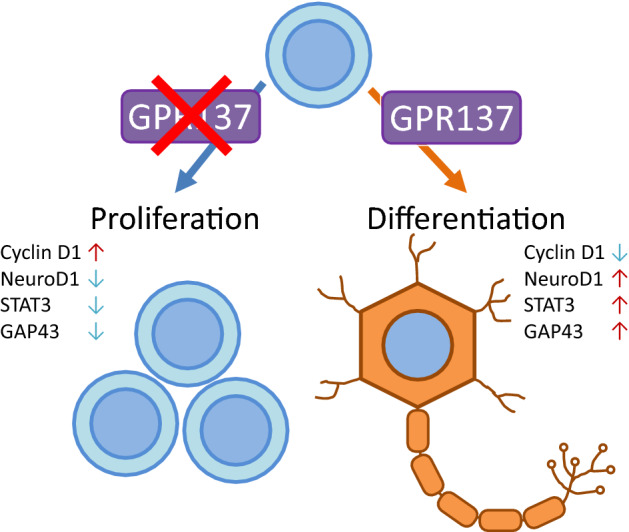

## Introduction

G protein-coupled receptor 137 (*GPR137*), discovered by searching the Genbank genomic database [1], is an orphan GPCR-encoding gene [2]. *GPR137 i*s also known as transmembrane 7 superfamily member 1-like protein, *C11orf4* or *GPR137A*, and is an integral membrane protein [3]. It is involved in the proliferation of tumor cells in several cancers, including ovarian [4], gastric [5], pancreatic [6], hepatoma [7], urinary/bladder [8], and prostate cancers [9], as well as medulloblastoma [10], malignant glioma [11], osteosarcoma [12], and leukemia [13]. RNA interference (RNAi)-mediated downregulation of *GPR137* inhibits tumor cell growth [5–7, 9–13]. These results indicate that *GPR137* plays a role in tumor cell proliferation and could be a potential therapeutic target for several types of cancers. In addition to tumor cells, *GPR137* is expressed ubiquitously, including in the central nervous system (CNS) [1]. However, the function of *GPR137* and its associated ligands in neuronal cells remains unknown.

Neuro2A cells are widely used as a neurite outgrowth model during neuronal differentiation [14] and share similar properties as neuronal progenitor cells (NPCs) [15, 16]. NPCs can proliferate a limited number of times and differentiate into neurons. The proliferative NPCs initially exist in an undifferentiated immature state and subsequently cease to proliferate and differentiate into mature neurons [17]. Cell cycle regulators and transcription factors are related to the differentiation of NPCs. Cyclin D1, a cell cycle regulator, promotes the transition from G1 to S phase and the progression of the cell cycle to maintain NPCs in an immature state [18]. The transcription factor, prospero homeobox protein-1 (PROX1), downregulates cyclin D1 expression [19]. Neurogenic differentiation factor 1 (NeuroD1) is a member of the basic helix-loop-helix (bHLH) protein family and plays a critical role in neuronal progenitors to neuronal differentiation [20]. Neurite outgrowth is a primary marker associated with neuronal differentiation, which is a crucial process in the development of neuronal functions. STAT3 is another critical transcription factor that promotes neurite outgrowth [21]. Growth-associated protein 43 (GAP43) is a neurite outgrowth marker and is usually expressed in differentiated neurons [22]. Signaling pathways such as cAMP response element-binding protein (CREB), protein kinase B (AKT), and extracellular signal-regulated kinase (ERK) play a vital role in NPC proliferation and differentiation [23, 24].

To evaluate *GPR137* function in neuronal differentiation, we established *GPR137* knockout (KO) neuro2A cells and investigated its role in neuronal differentiation.

## Materials and Methods

### Cell Culture

A mouse neuroblastoma cell line, neuro2A cells (IFO50081) were obtained from the JCRB Cell Bank (Osaka, Japan). The cells were maintained in Dulbecco’s Modified Eagle Medium supplemented with 10% (v/v) FBS and 1% penicillin–streptomycin (Invitrogen, Carlsbad, CA) in a humidified atmosphere containing 5% CO_2_ at 37 °C.

### GPR137 KO Neuro2a Cell Generation and GPR137 Genetic Rescue

Experimental protocols were approved by the DNA experiment safety committee of Saitama Medical University. *GPR137* KO neuro2A cells were generated using the Guide-it™ CRISPR/Cas9 systems (Takara Bio Inc., Shiga, Japan). *GPR137*-specific gRNAs (No.1 Forward: 5′-CCGGCTCTGGCCGACGCTTCGCCT-3′ Reverse: 5′-AAACAGGCGAAGCGTCGGCCAGAG-3′; protospacer adjacent motif (PAM) sequence; TGG: No.2 Forward: 5′-CCGGAGGCATCTAGCCGGCTCCGA-3′ Reverse: 5′-AAACTCGGAGCCGGCTAGATGCCT-3′; PAM sequence; GGG) were designed using CRISPR direct [25] and synthetic oligos were ligated into Guide-it-ZsGreen1 vector. The plasmid vectors were transfected into neuro2A cells with Lipofectamine 3000 (Invitrogen). Neuro2A cells expressing ZsGreen were selected and cultured as single cells by limited dilution. A Guide-it genotype confirmation kit (Takara Bio Inc.) was used to identify the homozygous mutants. In-del detection and cloning of targeting sites were performed using a Guide-it Indel Identification kit (Takara Bio Inc.). The colonies for KO were identified by the changes in their DNA sequences.

Rescue cells were then constructed to re-express *GPR137* in *GPR137* KO neuro2A (KO R) cells. The full open reading frame of murine *GPR137* complementary DNA (cDNA) was obtained by PCR with Pfu DNA polymerase (Promega, Madison, WI) from a cDNA library synthesized from murine mRNA using oligonucleotide primers (Forward: 5′-GAGGAAGAAGCCTCCCAATC-3′ and Reverse: 5′-CACCTGGGAGAAGAGCAGAG-3′). The PCR product was then ligated into pEF6/V5-His vector (Invitrogen). The rescue plasmid vectors were subsequently transfected into *GPR137* KO neuro2A cells with lipofectamine (Invitrogen). KO R cells stably expressing *GPR137* were selected and cultured as single cells by limited dilution.

### Reverse Transcription PCR (RT-PCR) and Quantitative Real-Time PCR (Q-PCR)

Total RNA was extracted from cells using ISOGEN (Nippon Gene, Tokyo, Japan) following the manufacturer’s instructions. Total RNA was reverse-transcribed using a PrimeScript RT reagent kit (Takara Bio Inc.). The following primer sequences were used for RT-PCR: *GPR137* (NO. 1 Forward: 5'-TGCTTCTGTATGGGCACAAG-3' and Reverse: 5'-CCCTATAGCAGCTGCCTGAC-3', No. 2 Forward: 5'-ATGCCAGCCGGGCCTGTTAC-3' and Reverse: 5'-AGCAGATCACGTCTGTGGTG-3').

Q-PCR was performed using the Quant Studio 12 K Flex (Applied Biosystems, CA). The following primer sequences were used: *Phosphoglycerate kinase 1* (PGK1; Forward: 5′-tgctgttccaagcatcaaa-3′ Reverse: 5′-gcatcttttcccttcccttc-3′); *Cyclin D1* (Forward: 5′-ttcagggaggaaatggactg-3′ Reverse: 5′-tccatgctgtcactctccag-3′); *PROX1* (Forward: 5′-cagcccgaaaagaacagaag-3′ Reverse: 5′-gcttgttctcagccatctcc-3′); *NeuroD1* (Forward: 5′-gatcaaaagcccaagagacg-3′ Reverse: 5′-gcgtctgtacgaaggagacc-3′); *STAT3* (Forward: 5′-gacccgccaacaaattaaga-3′ Reverse: 5′-tcgtggtaaactggacacca-3′); *GAP43* (Forward: 5′-ggctctgctactaccgatgc-3′ Reverse: 5′-ggcttgtttaggctcctcct-3′).

### Cell Growth Assay

Microculture tetrazolium technique (MTT) assay provides a quantitative measure of the number of viable cells by determining the amount of formazan crystals produced by metabolically active cells. Cells (1 × 10^5^ cells/well) grown in serum-containing medium in 24-well plates, were treated and 50 µl of MTT reagent [3-(4,5-dimethyl-2-thiazolyl)-2,5-diphenyl-2H-tetrazolium bromide] (FUJIFILM, Osaka, Japan) (5 mg/ml in in phosphate-buffered saline (PBS)) was added to each well. The plates were incubated in a humidified atmosphere of 5% of CO_2_ at 37 °C for 4 h. After removing the medium, formazan crystals were dissolved in 200 µl isopropanol/HCl (100: 0.34), and the absorbance was measured using a micro plate reader (Bio-Tek, Redmond, WA) at 570 nm relative to 630 nm. Data were normalized to the WT cells on day 1, and the means ± SEM of quintuple wells are expressed as percentages. Results are representative of three independent experiments.

### Measurement of Neurite Outgrowth

Cells (3 × 10^5^ cells/well) were seeded in 6-well plates and incubated for 24 h. The medium was then replaced by serum-free fresh medium with or without 10 μM retinoic acid (RA, FUJIFILM). After a 24 h incubation, 100 randomly selected cells in a well were photographed at 10 × magnification, and images were captured using a BZ-X710 microscope (Keyence, Osaka, Japan). The longest neurite lengths from a cell body and differentiated cells were measured, and the mean values per well were calculated. Differentiated cells were defined as cells with neurites longer than twice the cell body diameter [26]. Data are presented as means ± SEM of quintuple wells. Results are representative of at least two independent experiments.

### Western Blotting

Subconfluent cells were grown in serum-containing medium in a 10 cm dish. Cells were homogenized on ice in RIPA buffer [50 mM Tris–HCl pH 8.0, 150 mM NaCl, 5 mM EDTA, 1% NP-40, 0.1% SDS, 0.5% DOC] containing a protease inhibitor cocktail (Calbiochem, San Diego, CA) (1: 1000 dilution) with a tissue homogenizer (Brinkmann Instruments, Westbury, NY). Protein concentrations were determined using a bicinchoninic acid protein assay kit (Nacalai Tesque, Tokyo, Japan). Proteins (10 μg /lane) in lysates were separated by 12% SDS–polyacrylamide gel electrophoresis and transferred to nitrocellulose membranes (Bio-Rad, Redmond, WA). After blocking with 5% skim milk (Megmilk Snow Brand, Tokyo, Japan) in PBS containing 0.05% Tween 20 (polyoxyethylene sorbitan monolaurate, Nacalai Tesque) (PBS-T), the membranes were incubated with primary antibodies overnight, followed by incubation with horseradish peroxidase-conjugated secondary antibodies (Cell Signaling Technology, Beverly, MA) and then washing thrice with PBS-T. The membranes were then incubated with chemiluminescence reagent (Chemi-Lumi One Super, Nacalai Tesque; ImmunoStar LD, FUJIFILM). Images of the membranes were captured using a C-DiGit blot scanner (LI-COR, Lincoln, NE) and subjected to ImageJ analysis. Each membrane was probed with anti-GAPDH antibody (1: 1000, ABS16, Millipore, Billerica, MA), and the bands were used as loading controls. A pre-stained molecular weight marker was used to confirm expected sizes of the target proteins. Data were normalized to the WT cells, and the means ± SEM of quintuple dishes are expressed as percentages. Results are representative of three independent experiments.

The primary antibodies were anti-GPR137 (11929-1-Ab, Proteintech, Chicago, IL), anti-Phospho-Histone-H3 (PHH3, 66863-1-Ig, Proteintech), anti-caspase-3 (#9662, Cell Signaling Technology), anti-cyclin D1 (ab134175, Abcam, Cambridge, MA), anti-PROX1 (ab199359, Abcam), anti-NeuroD1 (ab213725, Abcam), anti-STAT3 (MAB1799, R&D Systems, Minneapolis, MN), anti-p-STAT3 (#9145 T, Cell Signaling Technology), anti-GAP43 (ab16053, Abcam), anti-CREB (ab32515, Abcam), anti-CREB1 (ab32096, Abcam), anti-AKT (#587F11, Cell Signaling Technology), anti-p-AKT (#9271S, Cell Signaling Technology), anti-ERK (#9102, Cell Signaling Technology), and anti-p-ERK (sc-7383, Santa Cruz, Dallas, TX). All antibodies were diluted 1: 1000.

### Statistics

Two-sample comparisons were carried out using a student’s *t*-test. Multiple comparisons were performed by one-way ANOVA followed by Newman-Keuls post-hoc test or two-way ANOVA followed by post-hoc Tukey test. All data were analyzed using Graph Pad Prism Ver. 5.01 (Graph Pad Software, Inc., San Diego, CA) and expressed as mean ± SEM. *p* values < 0.05 were considered statistically significant.

## Results

**Fig. 1:**
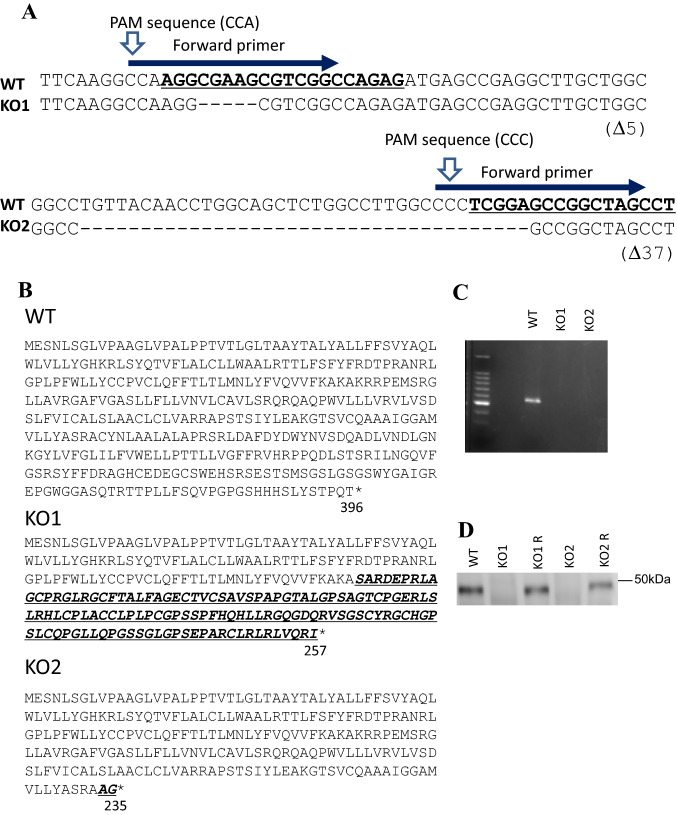
*GPR137* KO neuro2A cell establishment using CRISPR/Cas9 system. **A** Nucleotide sequences corresponding to *GPR137* and direct sequencing results of KO cells with their corresponding primers (indicated with blue arrows) and PAM sequences. **B** The amino acid sequence of the WT and CRISPR/Cas9-mediated *GPR137* genome editing. The frameshift mutation and premature termination observed in KO1 and KO2. **C** Gel electrophoresis analysis of the RT-PCR of *GPR137*. **D** Western blot analysis of the GPR137 protein for the WT, KO1, KO1 R, KO2, and KO2 R groups

*GPR137* KO neuro2A cells were generated using the CRISPR/Cas9 system with two gRNAs, and single cells were cloned. Targeting site cloning suggests that KO cells were homozygous mutants. Sequencing revealed a 5- and 37-base deficiency accompanying a frameshift in two strains (Fig. 1A). The amino acid changes were observed at positions 127 and 234 in KO1 and KO2, respectively (Fig. 1B). Premature terminations, i.e., the introduction of a stop codon, was observed at amino acid positions 257 and 235 in KO1 and KO2, respectively (Fig. 1B). We tested the mRNA expression of *GPR137* using reverse transcriptase polymerase chain reaction (RT-PCR) with primers specific to the deleted region. The amplification product of *GPR137* was observed in the wild type (WT) but not in KO1 and KO2 cells (Fig. 1C). These data confirmed that KO cells were successfully generated. Genetic rescue experiments were conducted by constructing the cells rescued to re-express *GPR137* in *GPR137* KO neuro2A (KO R) cells. The western blotting analysis confirmed that GPR137 protein was not expressed in KO1 and KO2 cells, whereas it was expressed in WT, KO1 R, and KO2 R cells (Fig. 1D).

**Fig. 2:**
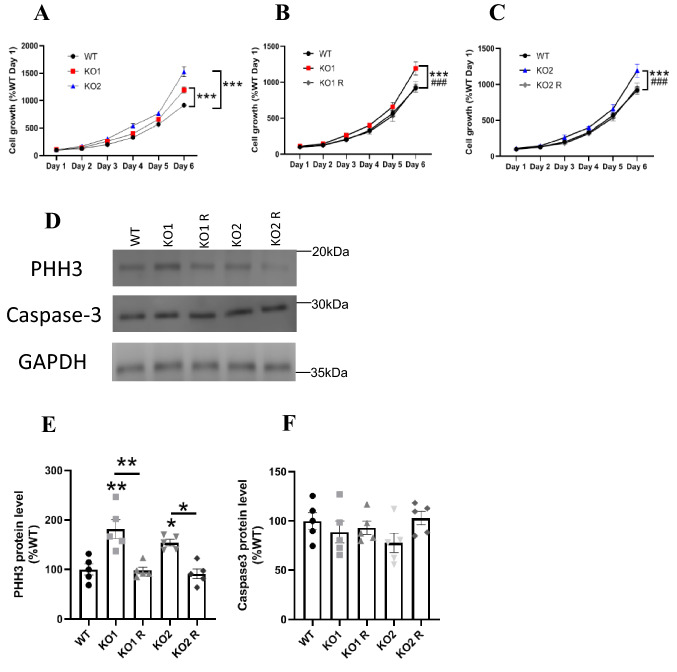
Cell growth of WT, *GPR137* KO neuro2A (KO) cells, and *GPR137* KO neuro2A + *GPR137* transfected (KO R) cells in serum contained medium. **A** Cell growth of the WT and KO cells. **B** Cell growth of WT, KO1, and KO1 R cells. **C** Cell Growth of WT, KO2, and KO2 R cells. Data are mean ± SEM, *n* = 5 per group. Statistical analysis was performed using two-way ANOVA followed by post-hoc Tukey test (***, ^###^*p* < 0.001). **D** Protein expression levels were determined by western blot analysis. Protein levels of PHH3 (**E**) and Caspase-3 (**F**). Data are means ± SEM, n = 5 per group. Statistical analysis was performed using one-way ANOVA followed by the post-hoc Newman-Keuls test (*p < 0.05; **p < 0.01)

We investigated the effect of *GPR137* deletion on cellular proliferation using the 2,5-diphenyl-2H-tetrazolium bromide (MTT) assay. KO1 and KO2 cells exhibited increased cell numbers compared to that in the WT (Fig. 2A). KO1 R and KO2 R cell numbers were comparable to that of the WT cells (Fig. 2B and C). PHH3 protein levels, a marker of proliferation, were increased in KO1 and KO2 cells (Fig. 2D and E). Caspase-3 protein levels, a marker of apoptotic, did not change following *GPR137* deletion (Fig. 2D and F).

**Fig. 3:**
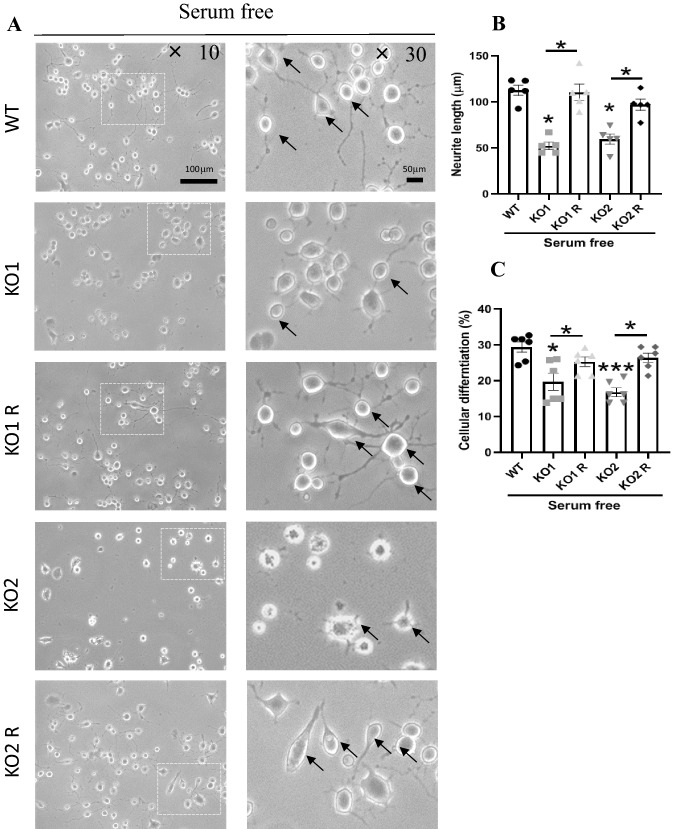
Cellular differentiation rates of the WT, *GPR137* KO neuro2A (KO) cells, and *GPR137* KO neuro2A + *GPR137* transfected (KO R) cells were evaluated following serum deprivation-induced neurite outgrowth. **A** Light micrographs of the differentiated cells. Black arrows indicate clearly differentiated cells. **B** Neurite length of WT, KO, and KO R cells. **C** Cellular differentiation rates of WT, KO, and KO R cells. Data are means ± SEM, n = 5 per group. Statistical analysis was performed using a one-way ANOVA followed by post-hoc Newman-Keuls test (*p < 0.05; ***p < 0.001)

**Fig. 4:**
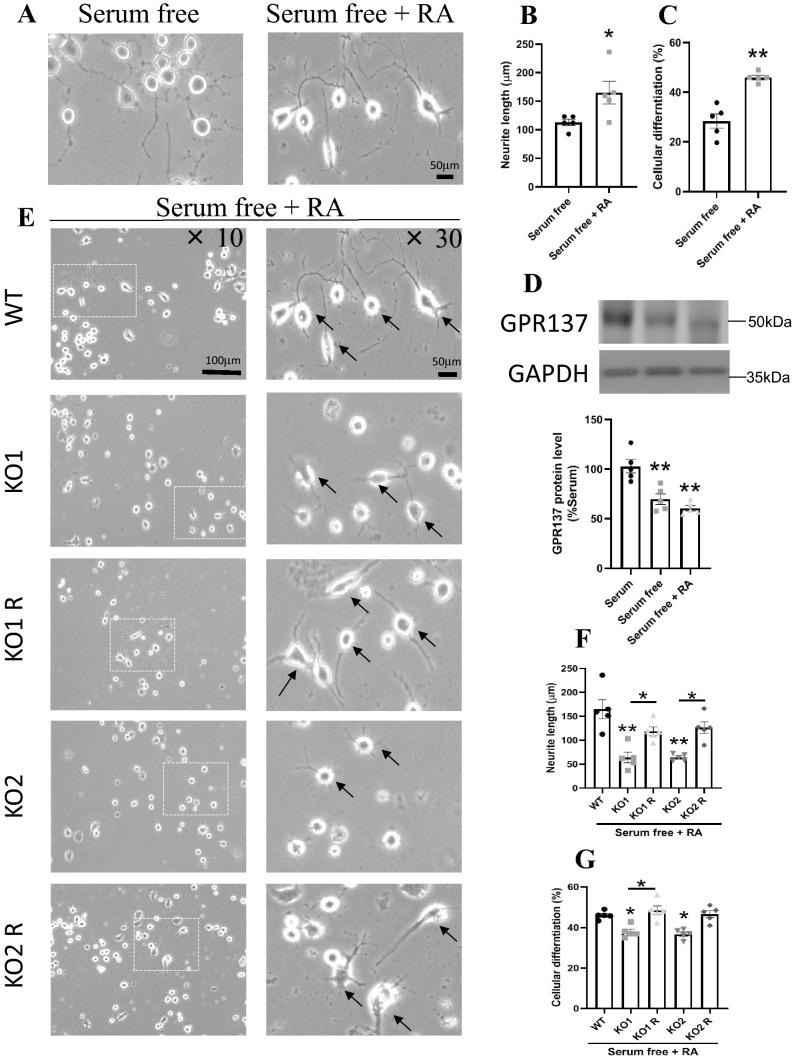
Cellular differentiation rates of WT, *GPR137* KO neuro2A (KO) cells, and *GPR137* KO neuro2A + *GPR137* transfected (KO R) cells were evaluated by serum deprivation-induced neurite outgrowth in the presence of retinoic acid. **A** Light micrographs of the differentiated cells by serum free and serum free with RA. **B** Neurite length of serum free and serum free with RA. **C** Cellular differentiation rates of serum free and serum free with RA. Data are presented as the means ± SEM, n = 5 per group. Statistical analysis was performed using a student’s t-test (*p < 0.05; **p < 0.01). **D** Protein levels of GPR137 by serum, serum free, and serum free with RA. **E** Light microscopic photographs of the differentiated cells. Clearly differentiated cells are indicated by black arrows. **F** Neurite length of WT, KO, and KO R cells. **G** Cellular differentiation rates of WT, KO, and KO R cells. Data are means ± SEM, n = 5 per group. Statistical analysis was performed using a one-way ANOVA followed by the post-hoc Newman-Keuls test (*p < 0.05; **p < 0.01)

To evaluate the effect of *GPR137* deletion on neuronal differentiation, we investigated the neurite outgrowth of neuro2A cells. Neuro2A cells respond quickly to serum deprivation, which induces neurite outgrowth [27]. Differentiated cells were characterized by neurites that were twice as long as the diameter of the cell body (Fig. 3A). WT cells exhibited normal neurite outgrowth, whereas KO1 and KO2 cells exhibited decreased neurite outgrowth (Fig. 3A and B). The neurite outgrowth levels in rescue cells, KO1 R and KO2 R, were similar to that in the WT cells (Fig. 3A and B). The WT cells induced approximately 30% differentiation. The percentages of differentiated cells were decreased in KO1 and KO2 cells compared to that in the WT cells (Fig. 3A and C). The percentages of differentiated cells were increased in KO1 R and KO2 R cells, similar to the level of WT cells (Fig. 3A and C). RA is a common inducer of neuronal differentiation [28]. We investigated neuronal outgrowth in the presence of RA. RA significantly increased neurite length and differentiation rates in neuro2A cells (Fig. 4A-C). The protein expression of GPR137 was decreased but was maintained in serum free and serum free with RA cells (Fig. 4D). KO1 and KO2 cells exhibited lower neurite outgrowth and differentiation rates, which were restored in KO1 R and KO2 cells (Fig. 4E-G).

**Fig. 5:**
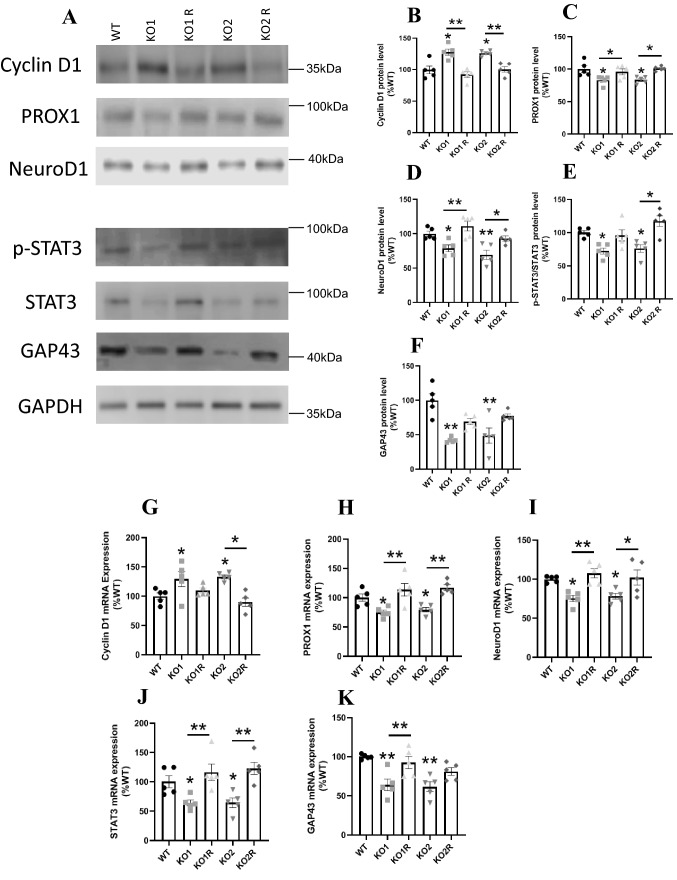
The expression of neuronal differentiation-related molecules in WT, *GPR137* KO neuro2A (KO) cells, and *GPR137* KO neuro2A + *GPR137* transfected (KO R) cells. **A** Protein expression levels were determined by western blot analyses. Protein levels of cyclin D1 (**B**), PROX1 (**C**), NeuroD1 (**D**), p-STAT3/STAT3 (**E**), GAP43 (**F**). The mRNA expression of cyclin D1 (**G**), PROX1 (**H**), NeuroD1 (**I**), STAT3 (**J**), GAP43 (**K**). Data are means ± SEM, n = 5 per group. Statistical analysis was performed using a one-way ANOVA followed by the post-hoc Newman-Keuls test (*p < 0.05; **p < 0.01)

**Fig. 6:**
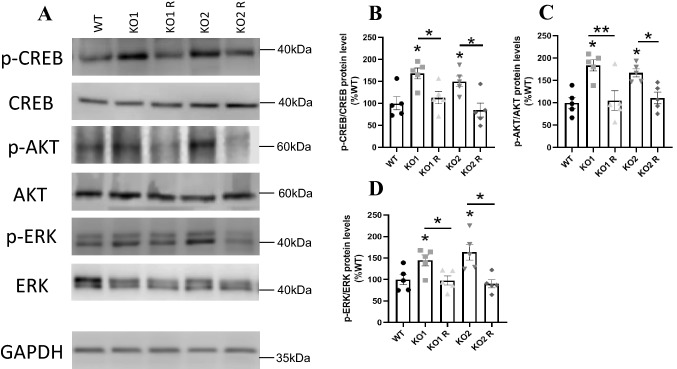
Intracellular signaling of WT, *GPR137* KO neuro2A (KO) cells, and *GPR137* KO neuro2A + *GPR137* transfected (KO R) cells. **A** Protein expression levels were determined by western blot analyses. p-CREB/CREB (**B**), p-AKT/AKT (**C**), and p-ERK/ERK (**D**). Data are mean ± SEM, n = 5 per group. Statistical analysis was performed using a one-way ANOVA followed by the post-hoc Newman-Keuls test (*p < 0.05; ***p < 0.001)

The effect of *GPR137* deletion on the expression of neuronal differentiation-related marker proteins, cyclin D1, PROX1, and NeuroD1, were investigated. Cyclin D1 expression levels were upregulated in KO1 and KO2 cells and were restored in KO1 R and KO2 R cells (Fig. 5A and B). PROX1 is a transcriptional factor that downregulates cyclin D1 [29], which was decreased in KO1 and KO2 cells, and restored in KO1 R and KO2 R cells (Fig. 5A and C). NeuroD1 expression was decreased in KO1 and KO2 cells and recovered in KO1 R and KO2 R cells (Fig. 5A and D). Phosphorylated STAT3 and GAP43 were downregulated in KO1 and KO2 cells and were restored in KO1 R and KO2 R cells (Fig. 5A, E and F). The gene expressions of these molecules were similar to the protein levels. The mRNA expressions of cyclin D1 were increased in KO cells (Fig. 5G). The mRNA expressions of PROX1, NeuroD1, STAT3, and GAP43 were decreased in KO cells (Fig. 5H–K). Phosphorylated CREB (Fig. 6A and B), AKT (Fig. 6A and C), and ERK (Fig. 6A and D) are upregulated in KO1 and KO2 cells and were suppressed in KO1 R and KO2 R cells.

## Discussion

To investigate the neuronal function of *GPR137*, we established *GPR137* KO neuro2A cells by CRISPR/Cas9-mediated genome editing. *GPR137* KO cells exhibited increased cellular proliferation and decreased neurite outgrowth, suggesting that *GPR137* has a role in cell cycle exit and neuronal differentiation in neuro2A cells. Moreover, these phenotypes were reversed in cells that were rescued to re-express *GPR137*. These data provide convincing evidence to support the function of *GPR137*.

We found that cell growth and PHH3 protein levels were increased, and caspase-3 levels were did not changed in *GPR137* KO cells. PHH3 is a marker of mitosis and cell proliferation [30], and caspase-3 is a marker of apoptotic cell death [31]. Elevated PHH3 protein indicated that *GPR137* KO cells underwent increased mitosis and cell proliferation. *GPR137* deletion did not affect caspase-3 protein levels, indicating that *GPR137* deletion did not suppress cell death. Thus, *GPR137* deletion increased cell proliferation without reducing cell death.

We demonstrated that the cyclin D1 protein expression was increased in *GPR137* KO cells, and was accompanied by a decrease of PROX1, a transcription factor that downregulates cyclin D1. Cyclin D1 directly regulates the immature state, and cell cycle acceleration and proliferation in NPCs [19, 32], whereas PROX1 suppresses neuro2A cell proliferation [29]. Furthermore, STAT3, CREB, and AKT signaling were increased in *GPR137* KO cells. STAT responsive elements [33, 34] and CRE [35] were identified in the cyclin D1 promotor. Therefore, STAT3 and CREB directly promote cyclin D1 transcription [33, 34, 36, 37]. Also, AKT upregulates cyclin D1 activity by preventing cyclin D1 proteolysis [38–41]. These results suggest that *GPR137* probably downregulate cyclin D1 by decreasing intracellular signaling via these pathways. Additionally, *GPR137* deletion decreased the neuronal differentiation marker, NeuroD1. NeuroD1 (also known as BETA2) plays a critical role in neuronal differentiation of NPCs [20] and induces cell cycle exit [42]. These results indicate that *GPR137* promotes cell cycle exit via cyclin D1 downregulation and neuronal differentiation, simultaneously upregulating NeuroD1.

*GPR137* involvement in neuronal differentiation was also revealed by decreased neurite outgrowth in *GPR137* KO cells. Moreover, the STAT3 and GAP43 protein levels were decreased in *GPR137* KO cells. STAT3 is also a key transcription factor that regulates neurite outgrowth in neuro2A cells [21]. GAP43 is expressed in the neurite growth cone and is a major determinant of neurite outgrowth [43]. Reduced neurite outgrowth and low marker protein levels suggest that neuronal differentiation is suppressed in *GPR137* KO cells. Therefore, these data also confirmed the role of *GPR137* in regulating neuronal differentiation.

CREB, AKT, and ERK signaling are involved in not only neuronal proliferation [44–46], but also differentiation [47–49]. Our results indicate that *GPR137* deletion increases the phosphorylation of CREB, AKT, and ERK, suggesting that *GPR137* downregulates the phosphorylation of these signaling pathways. Although further research is need to reveal which pathway is involved with neuronal differentiation mediated by *GPR137*, ERK signaling probably acts to enhance this process. This is supported by a previous study showing that activation of the ERK pathway stimulates neurite outgrowth in neuro2A cells [49, 50], which agrees with our results.

RA induces neuronal differentiation by activating the transcription of genes related to cell signaling, structure protein, enzymes, and receptors [51]. In this study, the phenotypes of cell with *GPR137* deletion were similar in the presence or absence of RA. We considered that the mechanism of *GPR137*-mediated neuronal differentiation was independent of RA signaling cascades.

Previous studies reported that *GPR137* plays a role in tumor cell proliferation [4–13]. In contrast, our results indicated that *GPR137* inhibits cell proliferation in neuro2A cells. Knockdown of *GPR137* downregulated the ERK and AKT pathways in osteosarcoma [12] and ovarian cancer cells [4], respectively. However, we found that *GPR137* deletion upregulated ERK and AKT signaling. These opposing effects of *GPR137* might be due to differences in *GPR137*-mediated signals between cancer and neuronal cells.

NPC proliferation is vital in maintaining the NPC pools during neurogenesis [52]. Subsequently, NPCs must halt their proliferation, accelerate cell cycle exit, and differentiate into neurons during brain development [17]. Regulation of these events by *GPR137* may be crucial in the formation of the neuronal structure.

## Data Availability

The data sets used and analyzed during the current study are available from the corresponding author on reasonable request.
